# HDR brachytherapy combined with 3D conformal versus IMRT in left‐sided breast cancer patients including internal mammary chain: Comparative analysis of dosimetric and technical parameters

**DOI:** 10.1120/jacmp.v6i3.2027

**Published:** 2005-08-17

**Authors:** Rajesh Ashok Kinhikar, Sudesh Sharad Deshpande, Umesh Mahantshetty, Rajiv Sarin, Shyam Kishore Shrivastava, Deepak Dattatraya Deshpande

**Affiliations:** ^1^ Department of Medical Physics Tata Memorial Hospital Mumbai 400012 India; ^2^ Department of Radiation Oncology Tata Memorial Hospital Mumbai 400012 India

**Keywords:** breast cancer, internal mammary chain, high dose rate, 3D conformal radiotherapy, intensity‐modulated radiotherapy, dose‐volume histogram

## Abstract

Treatment of the internal mammary chain (IMC) with radiation therapy (RT) for patients with breast cancer remains a controversial issue. Different treatment techniques have been proposed, including oblique electrons, electron‐photon combination, and partially wide tangents (PWTs). However, the residual heart dose can remain significant mainly for left‐sided lesions. With PWTs and intensity‐modulated radiotherapy (IMRT), respiratory movement and errors in IMC localization remain a problem. The goal of this paper is to evaluate the impact of IMC brachytherapy (IMCBT) combined with 3D conformal radiotherapy (3DCRT) planning on heart, lung, and contralateral breast doses compared with IMRT. All plans including IMCBT plus 3DCRT were done on PLATO; IMRT plans were generated using the Cadplan‐Helios inverse treatment‐planning software module with the “sliding window&rdquo; technique. Dose‐volume histograms (DVHs) were calculated for all volumes of interest. Conformity and homogeneity index was also calculated for the planning target volume (PTV). Dose distribution in the surrounding normal tissue was evaluated. The mean conformity of the PTV was found to be 1.06 with IMCBT plus 3DCRT and 1.12 with IMRT. The mean homogeneity (HI95/107) was found to be 1.4 with IMCBT plus 3DCRT and 3.32 with IMRT. Using the IMCBT plus 3DCRT technique, the mean dose to the heart, contralateral breast, ipsilateral lung, and contralateral lung decreased with values of 32%, 6.76%, 20% and 5.52%, respectively, compared with IMRT. This novel technique of IMCBT plus 3DCRT can potentially reduce the dose to the heart and lungs. In addition, it rivals IMRT because of its unique advantages in localization, obviating the need for respiratory gating and maximum sparing of heart and other structures.

PACS numbers: 87.53.Jw, 87.53.Kn, 87.53.Mr, 87.53, 87.53.Tf

## I. INTRODUCTION

Planning radiotherapy (RT) to the breast and regional nodes is challenging. Treatment of the internal mammary chain (IMC) with RT for patients with breast cancer remains a controversial issue. The role of IMC radiation is being evaluated in patients who are at high risk for IMC micrometastasis, that is, locally advanced breast cancer, with medial quadrant/central tumors >3 cm. It remains technically challenging to identify and irradiate the IMC homogeneously, while sparing organs at risk (OARs), such as heart, lungs, contralateral breast, and skin.^(^ ^1^ ^)^ Identification of internal mammary vessel is the best method for IMC localization.

Several techniques have been investigated to treat the IMC.^(^ ^2^ ^)^ The conventional approach involves both electron and photon fields, with a mixture of electron and photon beams typically being used to treat the IMC.^(^ ^3^ ^,^ ^4^ ^)^ To minimize the dose to the heart, some practitioners advocate the use of an abutting oblique electron field over the parasternal area to treat IMC.^(^ ^5^ ^)^ Planning studies^(^ ^6^ ^)^ reveal a high‐dose region of the order of 10% to 20% and a dose variability of up to 40%. Instead of treating the entire IMC, the use of partially wide tangents (PWTs) or wide split tangents has been described to cover the breast and superior IMC with a block placed to shield part of the heart.^(^ ^6^ ^)^

In recent years, great advances have been made in the delivery of RT with photons, intensity‐modulated radiation therapy (IMRT) being among the most promising techniques. Intensity‐modulated radiation therapy can result in homogenous dose distributions within complex target volumes while simultaneously sparing neighboring normal tissue. The use of IMRT for irradiation of the breast alone, without the regional nodes, has been described in the literature.^(^ ^7^ ^–^ ^11^ ^)^ Comparative planning studies have also been described using segmented IMRT versus non‐IMRT in the left breast and internal mammary lymph nodes elsewhere.^(^ ^12^ ^–^ ^16^ ^)^

There is no consensus over optimal IMCRT techniques because various techniques, such as electron‐photon, PWTs, and IMRT, differ in terms of dose to the IMC, heart, lungs, and breast. With PWTs and IMRT, respiratory movement, and errors in IMC localization remain a problem. Respiratory motion studies indicate that the heart moves away from the chest wall with deep inspiration.^(^ ^17^ ^)^ Simple maneuvers, such as holding one&apos;s breath at deep inspiration during part of breast radiation, have been used to improve treatment efficacy.^(^ ^18^ ^)^ Preliminary studies from several institutions have shown that it is possible to control patient breathing during RT delivery.^(^ ^19^ ^)^ However, the magnitude of this benefit on treatment efficacy and the logistics of incorporating breath hold on a large scale have not yet been established. There could be the risk of underdosing IMC due to inaccurate localization/respiratory movement.

Recently, a study was designed at our center to treat IMC with high dose rate (HDR) brachytherapy.^(^ ^20^ ^)^ During breast surgery, the medial end of ipsilateral second intercostals space was dissected, and internal mammary vessels (IMVs) were identified. The IMV (artery/vein) was looped, and with the principle of venesection, a 6F sterilized nylon tube (closed tip) was inserted to the fifth intercostal space, and the proximal open end of the catheter was brought out of the skin through a small incision in the first intercostal space. High dose rate brachytherapy gives inhomogenous dose distributions, resulting in sharp dose falloffs a few centimeters away from the implant, reducing the dose to the heart and adjacent lung. The IMV is routinely sacrificed for coronary bypass surgery, so IMV is dispensable. Hence, we propose this novel technique of IMV catheterization for IMCBT. Although high dose rates have been in clinical use with interstitial breast implants for several years, to our knowledge, HDRs have never been used in the treatment of IMC. Furthermore, no formal comparison of HDR with other treatment modalities for this indication has been published so far.

In the present study, investigations were done through comparative treatment planning to identify the potential improvements that could result from the use of IMC brachytherapy (IMCBT) plus 3DCRT for the treatment of IMC and left‐sided breast over IMRT. Therefore, the goal of this study was to evaluate the impact of IMCBT plus 3DCRT planning on heart, lung, and contralateral breast doses compared with IMRT.

## II. METHODS AND MATERIALS

The CT datasets from five patients with left‐sided breast tumors were used for comparison of different RT techniques. Orthogonal X‐rays were taken on the Ximatron simulator (Varian Medical Systems, Palo Alto, CA), and CT scans were obtained on CT‐Simulator (Somatom emotion, Siemens Medical Inc, Germany) to identify IMV. The scans included the entire lung in 8‐mm thick CT slices and extended approximately from the midclavicle to the upper abdomen. All the images were then transferred via DICOM to the PLATO treatment‐planning system (Nucletron Pvt Ltd, the Netherlands) for IMC brachytherapy plus 3DCRT planning and Cadplan (Varian Medical Systems, Palo Alto, CA) 3D treatment‐planning system for IMRT planning. The clinical target volume was defined as the ipsilateral intact breast and internal mammary nodal chain. The planning target volume (PTV) was defined as the clinical target volume plus a 5‐mm isotropic margin (except in the superficial direction, which was set to 0 mm) to account for setup uncertainties and patient movement. The OARs defined in all the contours were contralateral breast, heart, coronary region (antrolateral 2 cm^2^ of heart on axial CT scan), and lungs. To ensure that the volumes for a given patient were delineated in an identical fashion between the PLATO and Cadplan, contours were compared between the systems on a slice‐by‐slice basis.

### A. Treatment techniques

#### A.1 IMCBT combined with 3DCRT

IMCBT planning was done on PLATO‐Brachytherapy (v14.1.3). The catheter was reconstructed on each CT image. The target was only IMC. The average treatment length was 7 cm. The dosimetry was carried out with Ir^192^ stepping source. The step size used was 2.5 mm. The optimization was done on dose points and geometry. A total dose of 34 Gy in 10 fractions over 5 days (6 h apart) was delivered with microselectron HDR (Nucletron Pvt Ltd., the Netherlands), prescribed at 1 cm off‐axis (irradiating a cylinder with a diameter of 2 cm).

All patients later received 3DCRT to the left breast. The same CT dataset of a patient with left‐sided breast cancer provided for HDR brachytherapy was used for 3DCRT planning on PLATO (RTS v2.5.1). Two (nearly) parallel, opposed medial and lateral tangential beams were used with 6‐MV photon and 15° wedges. The wedge angles and the weights of the two tangential beams were optimized to obtain a homogeneous dose distribution in the central plane. The gantry angles were optimized manually to minimize the beam divergence along the dorsal beam edge to reduce irradiation of the heart and lungs. The isocenter was localized automatically to the PTV's center of mass. An isotropic margin of 7 mm around the PTV was used to define the field size and field shape (as seen from the beam's‐eye view) to account for the beam penumbra. Multileaf collimators (MLCs) projecting the leaf width of 5 mm at isocenter automatically shape the beam aperture. Three‐dimensional dose distribution was calculated with a grid space between 3.0 mm and 3.5 mm. The optimum universal wedges (15°/30°) were used. The planning system uses a convolution‐based pencil‐beam model^(^ ^21^ ^)^ with the equivalent tissue‐air ratio method^(^ ^22^ ^)^ for inhomogeneity corrections. The dose distribution was normalized to 100% at the isocenter in the central plane. The final plan was evaluated, ensuring a 95% isodose coverage of the PTV. A total dose of 50 Gy (2Gy/fraction, 5 days/week) for 5 weeks was prescribed at the isocenter.

The final and approved IMCBT and 3DCRT plans were loaded to the PLATO EVAL 2.9. The radiobiological corrections were applied in the radiobiology model available in the PLATO. The weight factor function is designed to mutually weight doses delivered by the RTS and BPS modalities for all dose fractions. A weight factor of one was considered for external beam as well as HDR brachytherapy. The value for α/β for the tumor was taken as 10. Symbol $\tau_{1/2}$ is the half lifetime of repair in the range 0.1 h to 10 h, and the value 0.5 was entered. Cumulative DVH was calculated for PTV and OARs. The volumes of the PTV and OARs were determined by a random sampling technique (PLATO EVAL 2.9).

#### A.2 IMRT planning

In addition to the IMCBT plus 3DCRT technique, inversely planned IMRT was performed with the “sliding window” technique on the same CT dataset. Intensity‐modulated radiation therapy plans were generated using the Cadplan‐Helios inverse treatment‐planning software module. Treatment planning was carried out for irradiation with 6‐MV photon beams formed by a MLC with a leaf width of 5 mm at the isocenter. For IMRT, five beams with gantry angles 30°, 75°, 125°, 290°, and 345° were used. The IMC was enclosed in the left breast and was set as a PTV. The goals of inverse treatment planning were optimum dose homogeneity within the target and minimal integral dose to OARs at the same time. Cumulative DVH was calculated for PTV and OARs. The tangential IMRT plans were also carried out.

### B. Evaluation and analysis

#### B.1 For PTV

After the calculation of the 3D dose distribution, isodose contours were displayed in all axial, frontal, and sagittal planes. Dose‐volume histograms were calculated for all volumes of interest. The mean dose of the cumulative DVH of the target volume (i.e., breast and IMC) in IMRT was used to evaluate dose distribution. Conformity^(^ ^23^ ^)^ and homogeneity^(^ ^24^ ^)^ for the PTV was calculated. Conformity index (CI) was defined as the ratio of prescription isodose volume to the target volume. Lower CI values correlate with better conformity. Homogeneity index ${(\text{HI}}_{95/107})$ was defined as the percentage of the PTV with a dose higher than 95% and lower than 107% of the prescribed dose.

#### B.2 For OARs

The mean dose was noted. In addition, the volume receiving more than 10 Gy, 30 Gy, and 45 Gy was noted.

## III. RESULTS

### A. For PTV

**Figure 1:**
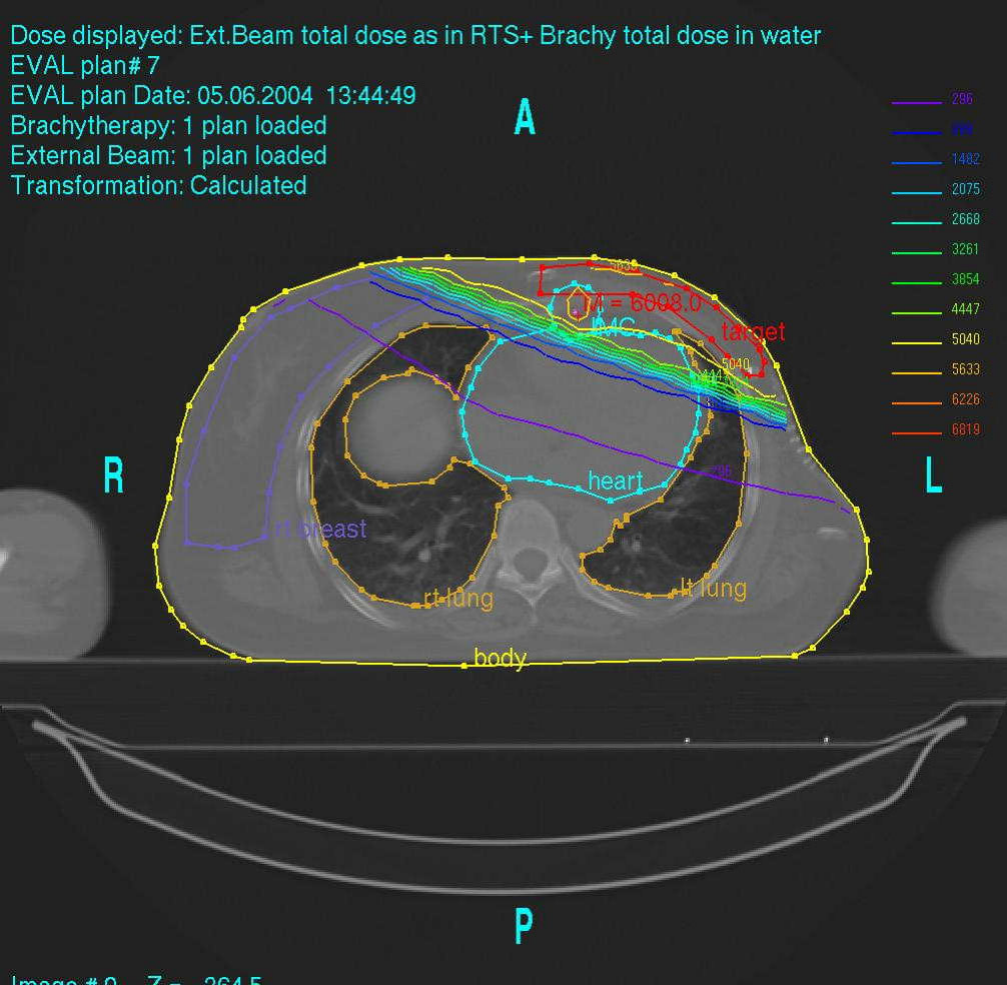
Isodose distribution in the axial plane for IMCBT plus 3DCRT with PLATO. Internal mammary chain was treated with HDR brachytherapy irradiating the 2‐cm cylinder; the left breast was treated with two tangential conformal 6‐MV photons with 15° wedges. The combined dose distribution has been taken from the PLATO EVAL module.

**Figure 2:**
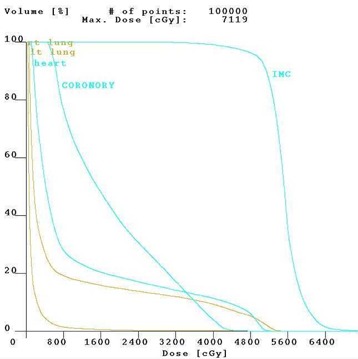
The cumulative DVH for the right lung, left lung, heart, coronary, and IMC for IMCBT plus 3DCRT from PLATO. Significant dose reduction of the organs at risks is seen.

**Figure 3:**
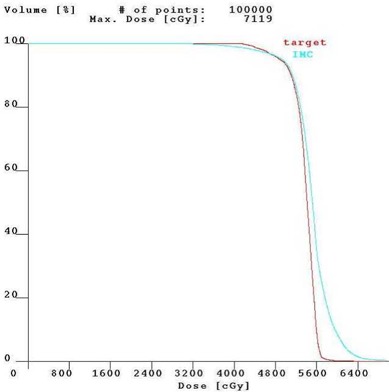
The cumulative DVH for target and IMC for IMCBT plus 3DCRT with PLATO

**Figure 4:**
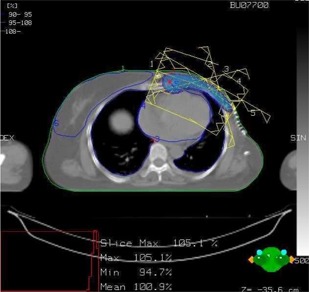
The isodose distribution in axial plane for IMRT with Cadplan. The typical IMRT plan shows five coplanar 6‐MV photon beams. The PTV includes left breast and IMC. The distribution shows 95% to 108% isodose lines around the PTV.

**Figure 5:**
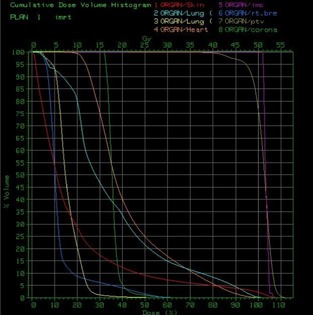
The cumulative DVH for PTV and the OARs for IMRT with Cadplan

Figure 1 shows the isodose distribution in the axial plane for IMCBT and 3DCRT in PLATO EVAL module. The contribution from both the external and brachytherapy is displayed. Internal mammary chain was the target for brachytherapy, while the left breast was the target for external. Internal mammary chain was treated with HDR, while the left breast was treated with two parallel‐opposed conformal tangential 6‐MV photon beams with 15° wedges. Figure 2 shows the integral DVH for heart, lungs, coronary, and left IMC for IMCBT and 3DCRT with PLATO. Figure 3 shows the integral DVH for PTV and IMC for IMCBT and 3DCRT with PLATO. Internal mammary chain, as well as the left breast, was covered with the 95% isodose. Figure 4 shows the isodose distribution in the axial plane for IMRT in Cadplan. The isodose washes of 90%, 95%, and 108% are shown. The PTV here was the IMC included in the left breast. Five coplanar beams were placed with 6‐MV photons. Figure 5 shows the DVH for PTV, heart and both lungs for IMRT in Cadplan.

The mean dose with IMCBT and 3DCRT to PTV was 100.5%. The mean dose with IMRT to PTV was 102.8%. The mean conformity of the PTV was found to be 1.06 with IMCBT and 3DCRT, and 1.12 with IMRT. The mean homogeneity ${(\text{HI}}_{97/107})$ was 3.32 with IMRT versus 1.4 with IMCBT and 3DCRT.

### B. For OARs

Table 1 shows mean doses and the volumes of the OARs receiving more than 10 Gy, 30 Gy, and 45 Gy, respectively, for plans with IMCBT and 3DCRT versus five fields IMRT. Table 2 shows the mean doses and the volumes of the OARs receiving more than 10 Gy, 30 Gy, and 45 Gy, respectively, for plans with IMCBT and 3DCRT versus tangential IMRT. To summarize, the IMCBT plus 3DCRT technique significantly reduces the doses to heart, both lungs, and contralateral breast. The mean dose to the coronary region was 21.1 Gy with IMCBT plus 3DCRT as compared to 38.52 Gy with IMRT.

**Table 1:** Average (over 5 patients) mean doses and the volumes of organs at risk receiving more than 10 Gy, 30 Gy, and 45 Gy. The range of the mean dose is shown in square brackets, and the range of standard deviation is also shown in parentheses.

Volume of interest	Description	IMCBT+3 DCRT	IMRT (5 fields)	Reduction in mean dose with IMCBT+3 DCRT
heart	Mean dose	7.58 [6.4–8.5]	23.44 [22.1–26.3]	32%
	(Gy)	(0.7008)	(1.368)	
		15.2%	99.7%	
	V10 (%)	8.9%	20%	
	V30 (%)	4.4%	5%	
	V45 (%)			
ipsilateral lung	Mean dose	4.86 [4.4–5.6]	24.3 [22.8–25.9]	20%
	(Gy)	(0.432)	(0.96)	
		20	94.4%	
	V10 (%)	5.14	25.7	
	V30 (%)	1.18	5.85	
	V45 (%)			
contralateral	Mean dose	0.48 [0.42–0.56]	8.7 [7.9–9.5]	5.52%
lung	(Gy)	(0.048)	(0.48)	
		4.72%	23.56%	
	V10 (%)	negligible	negligible	
	V30 (%)	negligible	negligible	
	V45 (%)			
contralateral	Mean dose	0.48 [0.45–0.52]	7.1 [6.4–8.5]	6.76%
breast	(Gy)	(0.02)	(0.6)	
		3.12%	17.25%	
	V10 (%)	negligible	negligible	
	V30 (%)	negligible	negligible	
	V45 (%)			

**Table 2:** Average (over 5 patients) mean doses and the volumes of organs at risk receiving more than 10 Gy, 30 Gy, and 45 Gy. The range of the mean dose is shown in square brackets, and the range of standard deviation is shown in parentheses.

Volume of interest	Description	IMCBT+3 DCRT	IMRT (Tangential)	Reduction in mean dose with IMCBT+3 DCRT
heart	Mean dose (Gy)	7.58 [6.4–8.5] (0.71)	14.46 [13.6–15.8] (0.99)	52.4%
	V10 (%)	15.2%	30%	
	V30 (%)	8.9%	17%	
	V45 (%)	4.4%	5%	
ipsilateral lung	Mean dose (Gy)	4.86 [4.4–5.6] (0.4)	11.04 [9.9–11.6] (0.7)	44%
	V10 (%)	20	20%	
	V30 (%)	5.14	12.5%	
	V45 (%)	1.18	5%	
contralateral lung	Mean dose (Gy)	0.48 [0.42–0.56] (0.05)	2.21 [1.9–2.5] (0.3)	21.7%
	V10 (%)	4.72%	5%	
	V30 (%)	negligible	Nil	
	V45 (%)	negligible	Nil	
contralateral breast	Mean dose (Gy)	0.48 [0.45–0.52] (0.02)	6.98 [6.5–7.3] (0.3)	6.87%
	V10 (%)	3.12%	10%	
	V30 (%)	negligible	5%	
	V45 (%)	negligible	2%	

## IV. DISCUSSION

In the present study we compared the IMCBT technique combined with 3DCRT and IMRT for multiple beams of left‐sided breast and the IMC. We emphasize that two different treatment‐planning systems were used, since brachytherapy calculation is not available in our Cadplan system. Both planning systems gave comparable volumes (<2% variation) and dose distributions. Both systems used a pencil‐beam algorithm for dose calculation. For inhomogeneity correction, PLATO used equivalent tissue air ratio, while Cadplan used modified Batho. PLATO and Cadplan used dose grids of 3.5 mm and 5 mm, respectively.

Each plan was studied carefully with IMCBT and 3DCRT before implementing it for clinical purposes. One of the important aspects of HDR brachytherapy planning is the rapid dose fall‐off at a few centimeters around the implant. Here, the DVH is calculated with respect to implant geometry. The organ‐based DVH could not be calculated at a larger distance from the implant since there will be a high dose around the catheter and a negligible dose at a distance of a few centimeters. When IMC brachytherapy is combined with the 3DCRT, the dose contribution to the contralateral breast and lung is primarily from the 3DCRT alone. There is a negligible contribution from HDR to these critical organs since they are quite far from the implant geometry and the target.

If the PTV includes only the breast, then the technique typically consists of two tangential fields placed medially and laterally to the breast. This field arrangement attempts to minimize the amount of underlying normal tissue irradiated. However, if the PTV also includes the IMC lymph node, then simple tangential fields usually do not offer the best solution. The inclusion of IMC creates an irregular concave volume difficult to irradiate adequately without delivering a significant dose to the heart, particularly in left‐sided breast cancer patients.

Because of the need to match the electron‐photon components, this technique usually provides poor target dose homogeneity. This dosimetric compromise is commonly accepted, however, to take advantage of the reduced dose to the ipsilateral lung and heart with the use of the electron field. But the reduction in heart dose can only be obtained at the expense of increased dose inhomogeneity, particularly along the match line between the medial tangential photon field and the abutting electron field. The main disadvantage of this technique is the significantly increased complexity of treatment planning and delivery.

Anatomically, the majority of the IMC lymph nodes lie superiorly, between the first and third intercostal spaces. With the oblique electron technique, the use of electrons minimizes the dose to the deeper structures, particularly heart and lungs. However, treatment planning and the delivery of the electron is more complicated. The junction between the photon and electron match line as well as the use of the anterior parasternal fields to irradiate the IMC contribute to increased dose inhomogeneity. In addition, the IMC depth determines the electron energy used, and this may limit its effectiveness in treating very deep IMC. The PWT technique improves dose homogeneity by avoiding field matching between the tangential and IMC fields. However, the residual heart dose can remain significant mainly for left‐sided lesions. Therefore, the need for technical improvements in RT delivery is obvious.

We have also planned the tangential IMRT to dosimetrically compare the organ doses with five‐field IMRT. The tangential IMRT plan has certainly reduced the doses to the critical organs. The wide split tangent technique can give better coverage of the breast but significantly overdoses the IMC. Although it is the simplest to plan and implement, the higher risk of complications suggests, at the very least, caution in its use. Moreover, to obtain complete target coverage with tangential fields, it is unavoidable to irradiate part of the ipsilateral lung. The dose to the lung should be as low as possible, to prevent radiation pneumonitis and late fibrosis.^(^ ^25^ ^)^ Dose analysis of the ipsilateral lung showed that the mean dose decreased from 24.3 Gy with IMRT to 4.86 Gy with IMCBT combined with 3DCRT. Consequently, the probability for inducing radiation pneumonitis was decreased for the IMCBT combined with 3DCRT technique.

A further impressive result of our present technique is the reduction of high dose area of a substantial cardiac volume containing major parts of the coronary vessels and the conducting system. IMCBT plus 3DCRT has the potential to overcome this limitation in treating deep IMC. It offered the best compromise between the two competing interests of the PTV (breast and IMC) and OARs. It was able to maintain low dose values for heart, both lungs, and contralateral breast.

The use of IMRT, however, requires significant resources and extensive quality assurance; it can also be time‐consuming to plan, verify, and deliver compared with other techniques. The IMRT plan was generated using a standardized set of parameters. At present, we do not offer IMRT to any patients with breast cancer when the IMC should be integrated into the target volume. There are several studies comparing irradiation of the left breast and IMC using conventional techniques with step‐and‐shoot IMRT. We have used the dynamic MLC “sliding window” technique for IMRT planning here.

Greater dose inhomogeneity in IMCBT combined with 3DCRT plan was caused by over‐dosage as opposed to underdosage. This is obvious because there is a high dose with brachytherapy around IMC irradiating a 2‐cm diameter. This high‐dose region is very close to the left breast, which is then treated with 3DCRT. But the percentage of overdose is only 2.17%, not very significant.

Another important concern over delivery of IMRT is the anticipated increased integral dose and volume of unspecified tissue receiving a low dose of radiation. It should be mentioned that the mean dose to both lungs, contralateral breast, and the integral dose to the whole body is increased by multibeam IMRT. The number of monitor units (MUs) to be delivered also increases in IMRT compared with 3DCRT. Total MUs delivered with IMRT ranged from 800 MUs to 900 MUs, compared with 250 MUs with 3DCRT. Before widespread application of inversely planned IMRT in breast cancer, the effect of low doses to an enlarged part of normal tissue should be evaluated in controlled clinical studies. The most sensitive structure for stochastic damage in the treated volume is the lung tissue. Here, IMRT delivers comparatively higher mean dose values. For the ipsilateral lung, an increase of the mean dose from 4.86 Gy to 24.3 Gy was obtained with IMRT.

One of the important aspects is the time required for the treatment planning and execution of IMRT. This also should be considered in the day‐to‐day machine workload. On average, it took 10 h to 15 h for IMRT planning, 4 h to 5 h for quality assurance, 10 min for patient setup with immobilization, and 20 to 25 min for the treatment. Most of the IMRT fields were split due to the limitation of the MLC carriage. IMCBT and 3DCRT planning took a maximum of 10 min and 30 min, respectively. It took 10 min each for IMCBT and 3DCRT actual treatment.

## V. CONCLUSIONS

The presented technique is safe, simple, and clinically feasible. Breast cancer patients could be irradiated with IMCBT combined with 3DCRT, thus reducing the dose to the heart and lungs, which is proposed as standard for this category of patients. IMCBT combined with 3DCRT gives satisfying dose distribution. This novel technique can potentially rival IMRT because of its unique advantages in localization, obviating the need for respiratory gating and maximum sparing of heart and other structures. Moreover, there is an increase in integral dose to the entire normal tissue with the application of IMRT. Internal mammary chain irradiation with the simple and safe technique of intra‐operative catheter placement could be an attractive alternative that not only ensures high dose to subcentimeter IMC nodes, but also confers maximum cardiac sparing.
